# Finite Element Analysis of Dental Implants with Zirconia Crown Restorations: Conventional Cement-Retained vs. Cementless Screw-Retained

**DOI:** 10.3390/ma14102666

**Published:** 2021-05-19

**Authors:** Jae-Hyun Lee, Ho Yeol Jang, Su Young Lee

**Affiliations:** 1 Department of Prosthodontics and Dental Research Institute, School of Dentistry, Seoul National University, 101, Daehak-ro, Jongro-gu, Seoul 03080, Korea; jaehyun.lee@snu.ac.kr; 2 Private Dental Clinic, 130 Cheongsa-ro, Seo-gu, Daejeon 35220, Korea; janghy5102@gmail.com; 3 Department of Prosthodontics, Seoul St. Mary’s Dental Hospital, College of Medicine, The Catholic University of Korea, 222 Banpo-daero, Seocho-gu, Seoul 06591, Korea

**Keywords:** abutment, dental implant, finite element analysis, stress, zirconia

## Abstract

The present study was designed to compare the stress distributions in two restoration types of implants and the surrounding bone. The first restoration type was a conventional cement-retained zirconia crown, and the second was a novel cementless screw-retained zirconia crown with a base abutment. A three-dimensional finite element method was used to model the implants, restorations, and supporting bone. A comparative study of the two implants was performed under two masticatory loads: a vertical load of 100 N and a 30-degree oblique load of 100 N. Under both loading conditions, the maximum von Mises stress and strain values in the implant and supporting bone were higher in the conventional cement-retained restoration model than in the cementless screw-retained model. In terms of stress distribution, the cementless screw-retained zirconia crown with base abutment may be considered a superior restoration option compared to the conventional cement-retained zirconia crown.

## 1. Introduction

The success of dental implants is based on the maintenance of the surrounding bone against stress [1]. The load transfers of the peripheral supporting bone around the implant may differ depending on the occlusal load direction, supporting tissue quality, implant specification, fixture surface, implant connection, and restoration-retaining type [2,3]. Determining the retaining type, such as screw- or cement-retained, is an important step in definitive restoration. This is because the marginal bone stability, success rates, and complication rates of implants can differ according to the retaining type of implant restorations [4].

Various features of screw- and cement-retained restorations have been reported in terms of passive fit, occlusion, retrievability, manufacturing convenience, economics, and maintenance of the supporting tissue [5,6]. One of the advantages of screw-retained prostheses is their retrievability; it is easier to detach the restoration from the fixture when necessary. They are also recommended for use in clinical situations with insufficient vertical space for restoration (less than 4 mm). Disadvantages include increased manufacturing time and expense and the presence of a screw-access hole on the occlusal surface [5,6]. The benefits of cement-retained restorations are the relatively easy fabrication procedure and passive fit between the abutment and implant. Their disadvantages include difficulty detaching prostheses, biological risk due to cement remnants, and unpredictable retention of cementation [6,7]. Thus, selecting the type of retention for implant prostheses is considered a clinically important decision.

The magnitude and patterns of stress in the restoration-implant–fixture-bone complex may be affected by the retention type of the implant-supported dental prosthesis [8,9]. Bone is a living dynamic tissue that undergoes remodeling by stimulation. If continuous overloading is applied to the peri-implant bone, bone resorption can occur, which could lead to implant failure [10,11,12,13]. Therefore, a biomechanical examination of the implant and surrounding bone is necessary to judge the success of the implant prosthesis by evaluating the dangers of marginal bone loss.

Recently, zirconia has become a popular material in the dental field because of its favorable physiochemical, optical, mechanical, and biocompatible characteristics [14,15]. Zirconia was introduced to dentistry in the early 1990s and has been extensively applied in the development of digital dentistry [15,16]. The successful use of zirconia for the fabrication of tooth-supported crowns and bridge restorations has attracted the attention of clinicians to expand their utilization of implant-supported prostheses [17]. However, little is known about how loads applied to zirconia prostheses are transmitted to the implant and bone under load.

**Figure 1:**
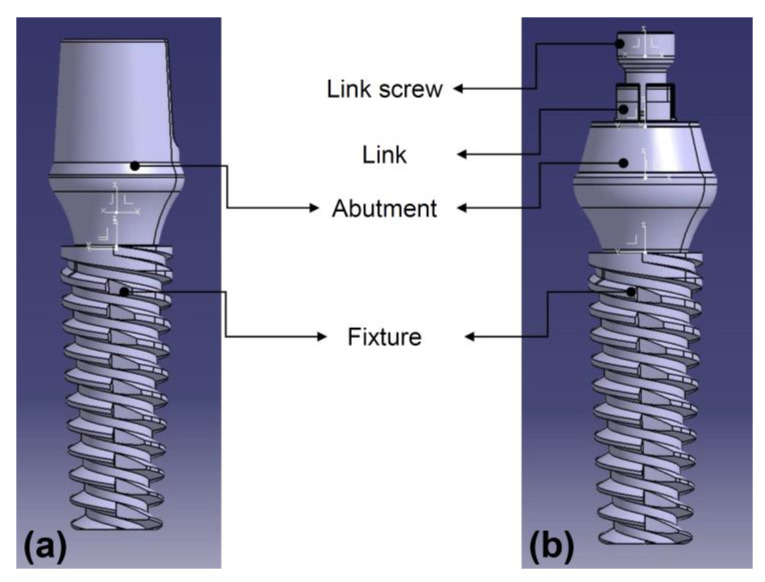
Schematic illustration of the prosthetic components used in this study. (**a**) Cement-retained and (**b**) cementless, screw-retained zirconia crown models.

Traditionally, when zirconia crowns are used for implant restorations, they are fabricated as cement-retained and cemented to a titanium abutment. Currently, a method of using a prefabricated titanium link is being increasingly implemented for fabricating screw-retained ceramic crowns. However, although this method can achieve chairside fabrication and retrievability of zirconia crowns, there are disadvantages, such as the crown falling off due to cement layer breakage and colonization of microorganisms in the cement layer. Recently, a method of fastening a zirconia crown to a titanium link without cement, in a “screw-in-screw” manner, has been developed. In this method, after a screw-type titanium base abutment is fastened to the implant, a titanium link and zirconia crown are fixed using a prosthetic (link) screw (Figure 1). This novel method eliminates the disadvantages of using cement in implant-supported zirconia crowns. Therefore, in this study, conventional cement-retained zirconia crowns and novel cementless screw-retained zirconia crowns were evaluated for stress and strain on the supporting implant and bone. The purpose of this study was to observe the stress and strain on the supporting structures of zirconia prostheses with two different types of retention during occlusal loading, using three-dimensional (3D) finite element analysis (FEA). The null hypothesis was that both retaining methods would produce similar stress and strain distributions in the implant and supporting bone.

## 2. Materials and Methods

A 3D model of the edentulous region of the lower second premolar area was prepared and the mandibular canal was removed to decrease the volume and complexity of the numerical model. The model simulates type II bone quality had a homogeneous cortical bone layer of 2 mm in thickness. The shape of the mandible segment was smoothed and rounded. Two 3D models were designed using solid modeling software (SolidWorks Corp., Concord, MA, USA): single implants with a conventional cement-retained zirconia crown and a cementless screw-retained zirconia crown.

Solid models of the mandible, implant, and zirconia crown were established from computed tomography data and managed using computer-aided design (CAD) in 3D FEA. Informatics programs were applied to generate a virtual 3D model. The reconstitution of the 3D model was performed using ANSYS Workbench^®^ (ANSYS Inc., Canonsburg, PA, USA). The analysis procedure included pre-processing for constructing the finite element model and post-processing for processing and representing the solutions [18,19].

The models aimed to clinically imitate the characteristics of implant prosthetic materials (Table 1). All analyzed information was transmitted to the ANSYS software (ANSYS Inc., Canonsburg, PA, USA), transferred from the physical condition to a mathematical model.

**Figure 2:**
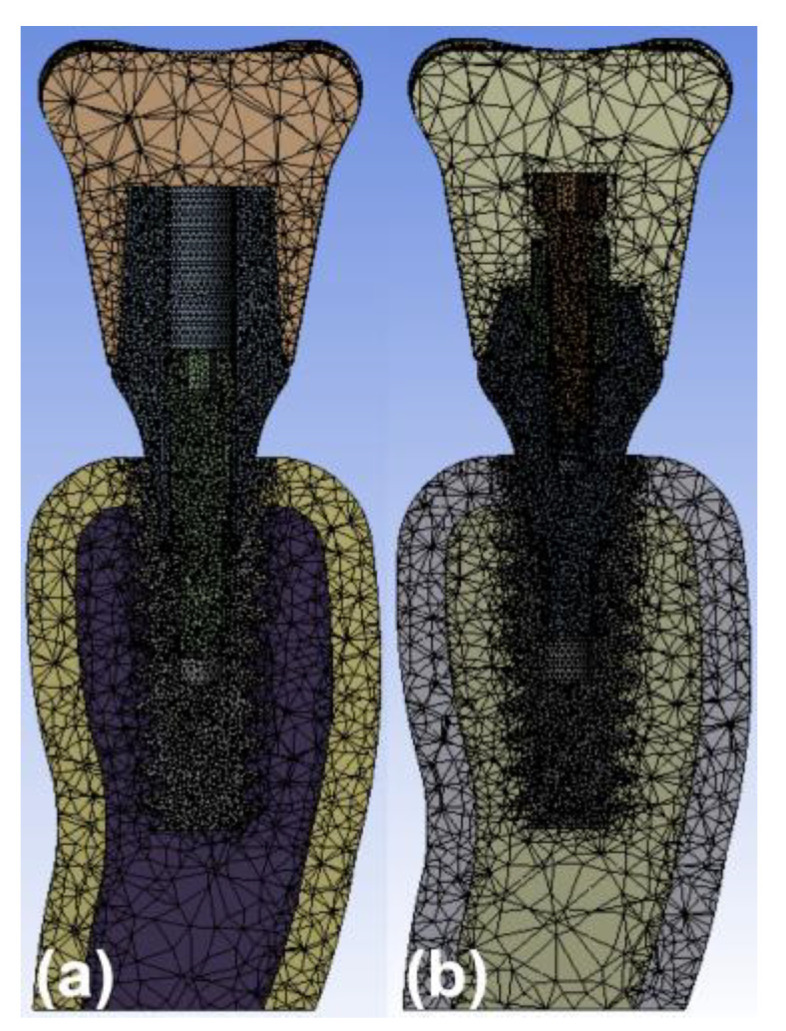
Meshes formed for the model with the properties of each component. The colors of cortical and cancellous bone are different, as are those of the implant and crown. (**a**) Cement-retained and (**b**) cementless screw-retained zirconia crown models.

The mesh was created using a digital software program (ANSYS 14, ANSYS Inc., Canonsburg, PA, USA). Figure 2 shows the meshes of the 2 models. The fixtures, abutments, abutment screws, crowns, cortical bone, and cancellous bone were regarded as homogeneous, isotropic, and linearly elastic. The implant was considered to be completely osseointegrated with the bone. The abutment was assumed to be in close contact with the implant. The surface between the screw and the implant and abutment was regarded as attached to the other surfaces. Overall, the completed models were meshed by parabolic tetrahedral elements. A coarse mesh of a typical element size of 0.6 mm was used for the crown. The screw was meshed with a typical element size of 0.2 mm. The same size was used for the implant. The mesh was refined near the neck of the implant to 0.1 mm. The model with screw-retained type incorporated 387,592 elements and 246,174 nodes, and the model with cement-retained type consisted of 316,058 elements and 213,152 nodes.

After acquiring these models, we performed FEA on the bone–implant–prosthesis using the ANSYS Workbench^®^ program (ANSYS Inc., Canonsburg, PA, USA) featuring a 2-way connected CAD with high productivity and a progressive design vision that combined the entire simulation procedure. A 3D linear model was constructed to represent the relationship (stress and strain) between the bone and implant fixture and between the abutment and abutment screw.

### 2.1. Choice of Materials

The implant components were designed as titanium alloys (Ti6Al4V). The characteristics of the materials were determined based on the modulus of elasticity, Poisson’s ratio, and density (Table 2).

### 2.2. Loading Conditions

**Figure 3:**
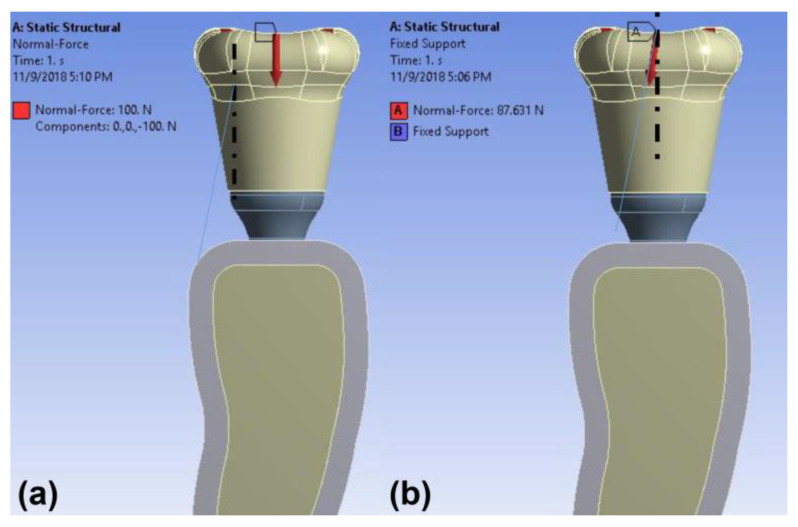
Schematic illustration of the loading conditions: (**a**) vertical and (**b**) oblique (30°) load.

The load simulation methods were identical for both models. An occlusal load of 100 N was applied to the crowns vertically and obliquely (30°) to the axis of the implants (Figure 3), based on previous studies [11,20]. The frictional coefficient of the surfaces was set to 0.3. The bone-implant contact was assumed to be fully osseointegrated.

### 2.3. Finite Element Analysis

After simulating the models with the ANSYS software (ANSYS WB 2.0 Framework, version 12.0.1, 2013 SAS IP), the values and patterns of the maximum von Mises stress and strain at the implant surface and surrounding bones were evaluated. The stress distributions were represented by color-encoded maps, with the highest stress in red and lowest in blue for the quantitative analysis.

## 3. Results

Table 3 and Table 4 list the maximum stress and strain values observed in the implant and surrounding bone according to the loading direction. Under both loading conditions, the maximum von Mises stress and strain values were greater in the cement-retained type than in the cementless screw-retained type. Additionally, a decrease in the stress in the bone was observed at points far from the implant. Moreover, the von Mises stress values were relatively higher for the oblique load.

### 3.1. Stress and Strain under Vertical Load

**Figure 4:**
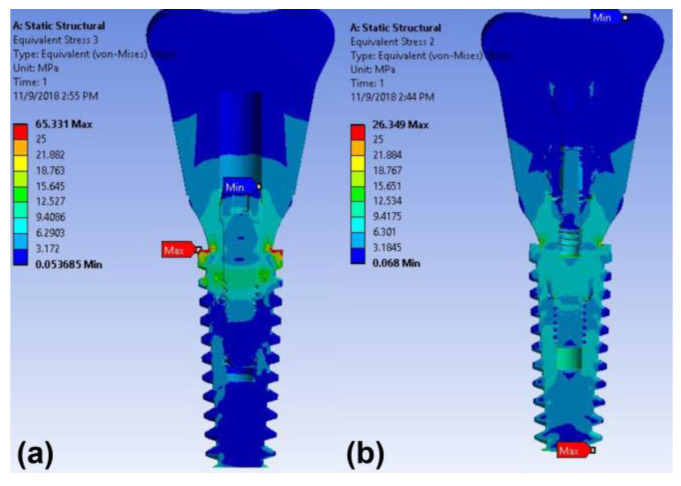
Color-coded von Mises stress distributions in the surrounding bone and implant under 100 N vertical load in the finite element model: (**a**) cement-retained and (**b**) cementless screw-retained zirconia crown models.

The maximum stress concentrations in the implant with the cement-retained zirconia crown and cementless screw-retained zirconia crown were detected around the neck and at the apical area of the implant under the 100 N axial load, respectively (Figure 4). The maximum von Mises stress value in the implant of the cementless screw-retained zirconia crown model (26.3 MPa) was less than that of the cement-retained model (65.3 MPa) under the 100 N vertical load (Table 3).

**Figure 5:**
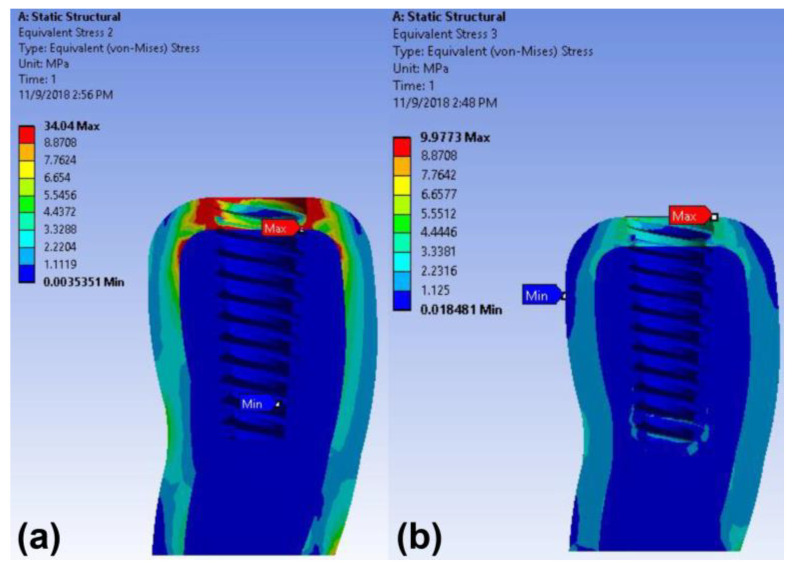
Color-coded von Mises stress distributions in the peri-implant bone under vertical load in the finite element model: (**a**) cement-retained and (**b**) cementless screw-retained zirconia crown models.

**Figure 6:**
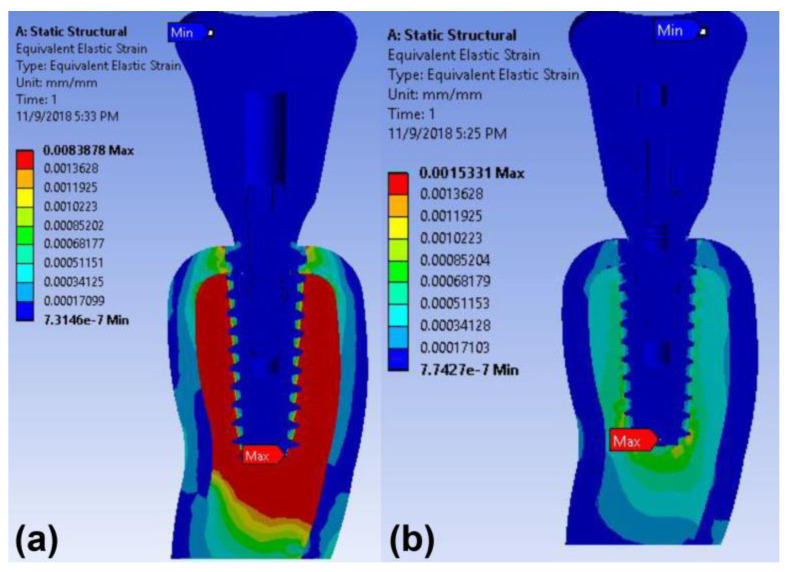
Color-coded strain distributions in the peri-implant bone under vertical load in the finite element model: (**a**) cement-retained and (**b**) cementless screw-retained zirconia crown models.

The maximum stress concentration on the peri-implant bone was found around the implant neck in both implant models under vertical loading (Figure 5). The maximum von Mises stress values in the cortical bone of the cementless screw-retained and cement-retained models were 9.97 MPa and 34.04 MPa, respectively (Table 3). The maximum strain value in the peri-implant bone of the cement-retained model was also higher than that of the screw-retained model under the axial loading (Table 3 and Figure 6).

### 3.2. Stress and Strain under Oblique Load (30 Degrees)

**Figure 7:**
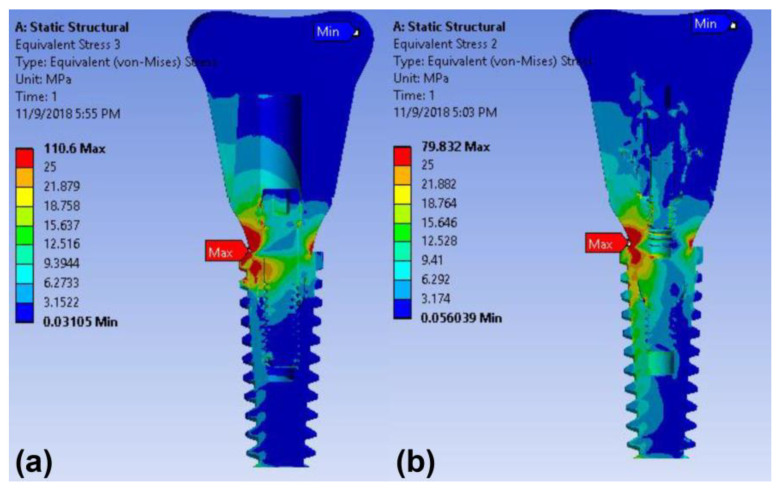
Color-coded von Mises stress distributions in the surrounding bone and implant under oblique load in the finite element model: (**a**) cement-retained and (**b**) cementless screw-retained zirconia crown models.

The maximum stress concentration in the implants was detected on the first and second threads of the implant in both models under the 30-degree oblique load condition (Figure 7). The maximum von Mises stress value in the implant of the cementless screw-retained model (79.83 MPa) was less than that of the cement-retained model (110.6 MPa) under oblique loading (Table 4).

**Figure 8:**
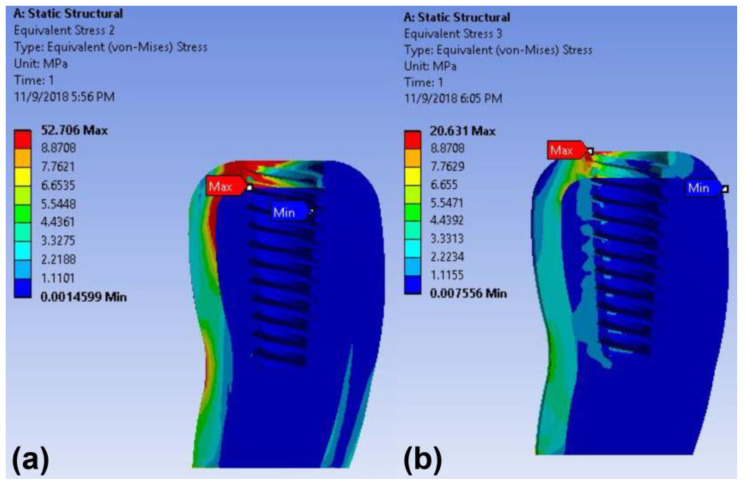
Color-coded von Mises stress distributions in the peri-implant bone under oblique load in the finite element model: (**a**) cement-retained zirconia crown model and (**b**) cementless screw-retained zirconia crown model.

In addition, the maximum stress concentration of the peri-implant bone was observed in the crest around the implants in both models under 30-degree oblique load (Figure 8). The maximum von Mises stress value in the peri-implant bone of the screw-retained model was 20.63 MPa, which was less than that of the cement-retained model (52.71 MPa) (Table 4).

**Figure 9:**
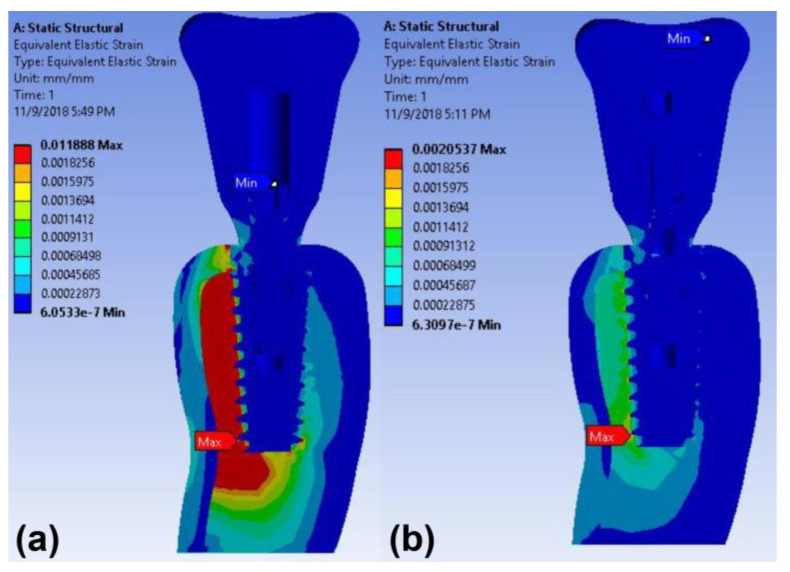
Color-coded strain distributions in the peri-implant bone under oblique load in the finite element model: (**a**) cement-retained zirconia crown model and (**b**) cementless screw-retained zirconia crown model.

The maximum strain concentrations in the peri-implant bone were detected around the apex area of the implant in both models under oblique loading (Figure 9). The maximum strain value in the bone was greater in the cement-retained type than in the screw-retained type (Table 4).

## 4. Discussion

The results of this study reveal that the retaining type of implant prostheses noticeably affects the stress and strain distributions in the implant components and surrounding bone. Thus, the null hypothesis of this study was rejected. The FEA showed that the dental implant model with a novel cementless screw-retained zirconia crown exhibited less strain and stress than the model with a conventional cement-retained zirconia crown.

In this study, the cementless screw-retained restoration had many separated components, including a titanium base abutment, titanium link, prosthetic (link) screw, and zirconia crown. Compared to the conventional cement-retained zirconia restoration, the cementless screw-retained type restoration had one more separated component. This difference between the models may explain why the stress and strain values in the surrounding bone and implant were higher in the cement-retained model than in the screw-retained model.

These results are consistent with those of previous studies [21,22]. A photoelastic stress analysis by Ochiai et al. [21] showed that non-segmented abutments under vertical loading generate more stress in the bone compared to segmented abutments. Another study suggested that segmented abutments show biomechanical benefits in terms of a decrease in stress concentration and microstrain in bone [22]. They found that a greater part of the stress might be detected in the prosthetic components (abutments and screws) before reaching the bone–implant contact area. This was explained by a previous report that the tolerance of the implant components can reduce the stress transmitted to the surrounding bone by allowing the components to move freely to some extent [23]. The conventional cement-retained restoration reduces the number of screws and micromovement of the components. Although this characteristic can be advantageous for reducing screw loosening and prosthetic component fracture, it may make it difficult to prevent the transmission of masticatory overload to the implant and supporting bone; the force applied to the occlusal surface may be transmitted directly to the bone rather than being dissipated by the prosthetic components. Therefore, these factors may increase the stress on the surrounding hard tissue.

In this study, the stress on the implant with the conventional cement-retained restoration was concentrated on the first and second threads of the implant. The maximum von Mises stress of the implant was observed in the implant neck for the conventional cement-retained zirconia crown. This result is in accordance with previous studies [10,11,24]. When the stress generated in the alveolar crest surpasses the elasticity of the bone, it causes microcracks in the bone and leads to marginal bone loss [25]. To prevent this complication, a favorable quality of bone must exist around the collar area of the implant [26]. However, in this study, the stress on the cementless screw-retained restoration was distributed throughout the implant and prosthetic components, rather than concentrating at the implant neck. The maximum stress concentration was detected in the apical area of the implant under the 100 N axial load (Figure 4). This result indicates that the stress spread of the cementless screw-retained prostheses with multi-components is more favorable than that of the implant model with the cement-retained restoration.

In addition, the present study found greater stress concentration under oblique loading than under vertical loading, regardless of the retaining type of the implant restoration. These results are consistent with those of previous reports [27,28]. Thus, fabricating a crown with a lower cusp slope is advantageous for decreasing oblique overload and diminishing the generation of deformation.

Through the use of FEA, we were able to numerically determine how much stress and strain was delivered by the cementless screw-retained prosthetic system to the implant and supporting bone. However, owing to the limitations of the finite element methodology, the models used in this study did not accurately represent the actual oral conditions of humans. Cortical and cancellous bones both react differently to load/unload cases with respect to the monotonic load case. There could be damaged zones or load redistributions. Thus, further clinical studies are required to validate the results of the present study.

Within the limitations of this FEA, the novel cementless screw-retained zirconia restoration transmitted less stress and strain to the implant and surrounding bone than the conventional cement-retained zirconia restoration. A cementless screw-retained zirconia crown with multi-prosthetic components might be a better choice than a conventional cement-retained zirconia crown in terms of stress distribution.

## Figures and Tables

**Table 1:** Specification of the components used in the study.

Component	Cement-Retained Model	Screw-Retained Model
Fixture	4.5 × 11 mm	4.5 × 11 mm
Abutment	5.5 × 11.25 mm	5.7 × 10.7 mm
Link	–	4.3 × 3.5 mm
Screw	2.3 × 9.5 mm	2.3 × 8.5 mm
Crown	8.0 × 10 mm	8.0 × 10 mm

**Table 2:** Material properties used in the finite element analysis.

Component	Modulus of Elasticity (GPa)	Poisson’s Ratio (ν)	Density (kg/m^3^)
Cancellous Bone	1.3	0.30	500
Cortical Bone	13	0.30	1180
Titanium (Fixture, Abutment, Link, Screw)	103	0.33	4620
Zirconia (Crown)	200	0.31	6090

**Table 3:** Maximum von Mises stress and strain values in the implant and surrounding bone under 100 N vertical load in the finite element model.

Component	Cement-Retained Model	Screw-Retained Model
Implant (stress)	65.3 MPa	26.3 MPa
Bone (stress)	34.04 MPa	9.97 MPa
Bone (strain)	0.0084 mm/mm	0.0015 mm/mm

**Table 4:** Maximum von Mises stress and strain values in the implant and surrounding bone under 100 N oblique load in the finite element model.

Component	Cement-Retained Model	Screw-Retained Model
Implant (stress)	110.6 MPa	79.83 MPa
Bone (stress)	52.71 MPa	20.63 MPa
Bone (strain)	0.012 mm/mm	0.002 mm/mm

## Data Availability

The data presented in this study are available on request from the corresponding author.
